# Life-Threatening Haemodynamic Instability During General Anaesthesia in a Child With Renal Tubular Dysgenesis: A Case Report

**DOI:** 10.7759/cureus.100080

**Published:** 2025-12-25

**Authors:** Shizuka Nohara, Satoshi Aoki, Taiki Kojima

**Affiliations:** 1 Anesthesiology, Aichi Children's Health and Medical Center, Obu, JPN

**Keywords:** general anaesthesia, hypotension, renal tubular dysgenesis, renin-angiotensin system, vasopressin

## Abstract

Autosomal recessive renal tubular dysgenesis (RTD) is attributed to a rare genetic mutation affecting the renin-angiotensin system, leading to reduced production and activity of angiotensin II. Because only a small number of patients survive beyond the neonatal period, information on safe anaesthetic management remains scarce. This report describes the case of a seven-year-old girl with autosomal recessive RTD who developed life-threatening hypotension requiring cardiopulmonary resuscitation immediately after induction of anaesthesia for surgery. When she was rescheduled for surgery a year later, we maintained a stable haemodynamic status by perioperative administration of fluids and careful administration of anaesthetics and vasopressors during general anaesthesia. Anaesthetists should be aware of the potential for life-threatening hypotension during general anaesthesia in children with RTD. General anaesthesia in these children requires detailed preoperative planning with adequate peri-anaesthetic fluid administration, careful titration of anaesthetics, and the use of vasopressors to prevent lethal hypotension.

## Introduction

Autosomal recessive renal tubular dysgenesis (RTD) is a rare disorder characterized by proximal tubular hypoplasia due to genetic mutations involving the renin-angiotensin system. RTD can prove fatal during the neonatal period, when infants often develop anuria, refractory arterial hypotension, and hypoxia as a result of pulmonary hypoplasia [1]. RTD is an extremely rare and sporadic disorder with approximately 150 documented cases in the literature, of which only 18 patients have survived beyond the neonatal period. Because most affected fetuses or neonates die shortly after birth, the true incidence of RTD remains unknown.

The primary pathophysiology in RTD is reduced production and activity of angiotensin II (ATII), which occurs because of genetic mutations in the renin-angiotensin system and decreased release of aldosterone and vasopressin [2-4]. Clinicians should be vigilant for potentially life-threatening hypotension during anesthesia in these patients, given that RTD carries substantial anesthetic risks such as pronounced vasodilation and severe hypotension. However, there is a lack of evidence on anesthesia management in RTD, which can be explained by the rarity of survival beyond the neonatal period.

We encountered a child with RTD in whom surgery had to be abandoned because of life-threatening circulatory collapse requiring cardiopulmonary resuscitation immediately after initiating induction of general anesthesia. One year later, the same child was scheduled for surgery of a ventriculoperitoneal (VP) shunt extension again. We maintained stable circulation during general anesthesia in this child by adequate peri-anesthetic fluid administration, careful titration of anesthetics, and vasopressors. This report summarizes two anesthetic events in the same patient and discusses anesthesia management and hemodynamic control in children with RTD.

## Case presentation

The patient was a seven-year-old girl who had weighed 953 g when she was delivered via cesarean section at 27 weeks. She was noted to have anuria and hypotension (mean blood pressure (BP) <15 mmHg) immediately after birth. On day 6 of life, the patient developed an intracranial hemorrhage, which led to hydrocephalus, and a VP shunt was placed at four months of age. Aside from findings directly attributable to RTD, such as oligohydramnios, severe neonatal hypotension, renal dysfunction with nonspecific findings on renal ultrasound, and hypoaldosteronism, no additional congenital anomalies were identified. The patient exhibited severe hypotension (systolic blood pressure was under 50 mmHg) at the time of this surgery, but it was interpreted as being due to extreme prematurity. She did not experience major anesthetic complications, although renal dysfunction persisted for several weeks postoperatively. RTD was diagnosed when genetic testing identified a heterozygous mutation in the angiotensin-converting enzyme (ACE) gene at seven months. At 13 months of age, the patient underwent gastrostomy placement, during which she also developed marked hypotension under general anesthesia; however, anesthesia was successfully managed through careful adjustment of sedative and analgesic agents. Since she had chronic renal failure and nephrogenic diabetes insipidus secondary to RTD, persistent polyuria required strict fluid management via gastrostomy, intravenous, and oral routes, as well as meticulous control of electrolytes, including potassium and sodium. After fludrocortisone was initiated at two years of age, her urine output stabilized, and rigorous fluid balance management was continued thereafter.

First anesthesia

At the age of seven years (height 108 cm, weight 19.3 kg), general anesthesia was scheduled for a VP shunt extension due to insufficient shunt length resulting from growth. She was taking fludrocortisone 0.12 mg/day preoperatively to maintain her serum sodium concentration. Preoperative nil per os status was initiated nine hours before surgery, at which time 100 mL/h of 0.9% sodium chloride with 5% glucose was started via gastrostomy and continued until two hours before surgery. No perioperative steroid coverage was performed.

Inhalational induction was initiated using 40% nitrous oxide with oxygen and 8% sevoflurane. Four minutes after induction, the pulse oximetry waveform disappeared, and there was no palpable pulse. Cardiopulmonary resuscitation was initiated, and tracheal intubation was performed with a 4.5-mm cuffed tracheal tube. Repeated bolus administration of 10 μg/kg epinephrine was required. Twenty-five minutes after initiating a continuous 0.10-0.25 μg/kg/min infusion of epinephrine, her vital signs stabilized (BP 80/50 mmHg, heart rate (HR) 157 bpm). Because it was considered that awakening the patient would contribute to hemodynamic stabilization, sedative agents were discontinued, and she was extubated in the operating room. Time under general anesthesia was 90 minutes. The surgery was abandoned, and the patient was admitted to the pediatric intensive care unit (PICU).

**Figure 1:**
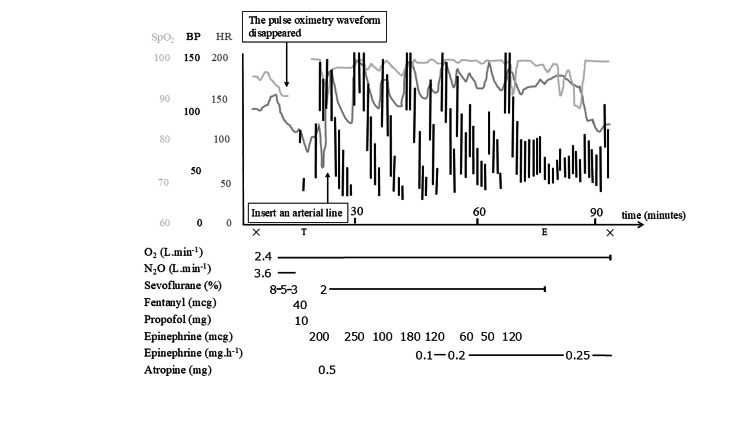
Hemodynamic changes during the first anesthesia. Symbols: T, intubation; E, extubation; ◎, start and end of surgery

Immediately after the event, she developed acute kidney injury with a serum creatinine level of 2.47 mg/dL. However, renal function gradually improved with careful adjustment of catecholamine support and fluid administration. She was moved to the ward on postoperative day (POD) 3 and discharged home on POD 10 with no abnormal neurological findings (Figure 1).

Second anesthesia

At the age of eight years (height 115 cm, weight 21.7 kg), the patient was rescheduled for VP shunt extension surgery. Preoperatively, the patient was on fludrocortisone 0.2 mg/day and desmopressin 360 μg/day. She was fasted for 12 hours before surgery, during which an 80 mL/h intravenous infusion of sodium chloride and Ringer’s acetate with glucose 5% was administered. Intravenous hydrocortisone succinate 85 mg (100 mg/m^2^) was administered as preoperative steroid coverage.

**Figure 2:**
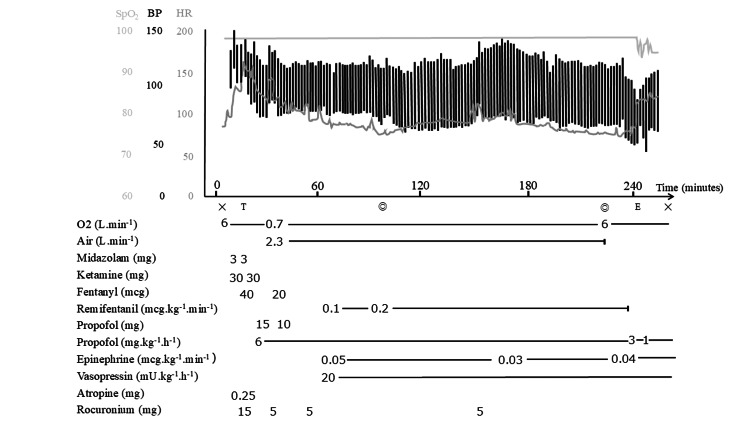
Hemodynamic changes during the second anesthesia. Symbols: T, intubation; E, extubation; ◎, start and end of surgery

Anesthesia induction was initiated with careful titration of midazolam, ketamine, and fentanyl (with respective total doses of 0.3 mg/kg, 3 mg/kg, and 3 μg/kg). Tracheal intubation was performed with a 5.5-mm cuffed tube, and an arterial pressure line was placed at the left ulnar artery. An intraoperative infusion of Ringer’s acetate with glucose 1% (200 ml/h) was started upon arrival in the operating room. The patient’s hemodynamic status was stable (BP 137/105 mmHg, HR 142 bpm) during anesthesia induction (Figure 2).

General anesthesia was maintained with propofol 6 mg/kg/h, remifentanil 0.1-0.3 μg/kg/min, and intermittent boluses of rocuronium 0.5 mg/kg and fentanyl 1 μg/kg under bispectral index monitoring. Hemodynamic stability was maintained (BP 120/60 mmHg, HR 75 bpm) by administering epinephrine 0.03-0.05 μg/kg/min and vasopressin 0.33 mU/kg/min during the surgery (Figure 2). Time under general anesthesia was four hours and 27 minutes, with an operating time of two hours and 13 minutes. The intraoperative fluid infusion volume was 504 mL, intraoperative urine output was 400 mL, and intraoperative blood loss was 7 mL. Blood loss was calculated by subtracting irrigation volume from suction canister volume and by weighing surgical gauze. The patient was admitted to the PICU and discharged to the general ward on the day of surgery. Fludrocortisone and oral desmopressin were resumed on POD1, and the patient was discharged without complications on POD14.

Table 1 shows the comparison of the first and second anesthesia.

**Table 1:** Comparison of the two anesthetic events. Abbreviations: BP, blood pressure; HR, heart rate; PICU, pediatric intensive care unit; POD, postoperative day

Parameter	First anesthesia (age 7)	Second anesthesia (age 8)
Indication for surgery	VP shunt extension	VP shunt extension
Preoperative medications	Fludrocortisone 0.12 mg/day	Fludrocortisone 0.2 mg/day, desmopressin 360 µg/day, hydrocortisone 85 mg IV pre-op
Preoperative fluid management	100 mL/h 0.9% NaCl + 5% glucose via gastrostomy until two hours before surgery	80 mL/h NaCl + Ringer’s acetate with glucose 5% IV until surgery
Preoperative urine output	Not measured	Not measured
Preoperative serum sodium/potassium	140 /4.2	140/3.9
Preoperative specific gravity	Below the detection limit	1.013
Perioperative BUN	31.7 → 72.1	11.5 → 10.3
(mg/dL)		
RPA/ aldosterone(pg/mL)/	Not measured	0.7/4.0
Perioperative serum creatinine (mg/dL)	0.69 → 2.47	0.48 → 0.53
Fasting duration	9 hours	12 hours
Induction method	Inhalational: 40% N₂O + 8% sevoflurane, fentanyl (2 µg/kg)	Intravenous: titrated midazolam (0.3 mg/kg), ketamine (3 mg/kg), fentanyl (3 µg/kg)
Intubation	Cuffed tube 4.5 mm	Cuffed tube 5.5 mm
Hemodynamic	Severe hypotension →	Hemodynamically stable:
	Pulselessness within 4 min	BP 137/105 mmHg, HR 142 bpm
Response during induction	CPR + repeated epinephrine boluses (10 µg/kg)	Not required
Vasopressor use (intraoperative)	Epinephrine infusion 0.10–0.25 µg/kg/min	Epinephrine 0.03–0.05 µg/kg/min + vasopressin 0.33 mU/kg/min
Anesthetic maintenance	Not performed (procedure aborted)	Propofol 6 mg/kg/h +
		Remifentanil 0.1–0.3 µg/kg/min + rocuronium/fentanyl boluses
Intraoperative fluid	Not measured	Ringer’s acetate with 1% glucose (200 mL/h)
Urine output	Not measured	400 mL
Estimated blood loss	0 mL (surgery aborted)	7 mL
Surgical outcome	Surgery aborted; PICU admission	Procedure completed; stable postoperative course
Postoperative course	PICU → ward on POD 3 → discharge POD 10	PICU same day → ward → discharge POD 14

## Discussion

We encountered a child with RTD who showed hemodynamic collapse after initiating an induction of inhalational anesthesia. Subsequently, careful perioperative optimization of the patient’s fluid status, vasopressin administration, titration of the anesthetics used for induction, and intraoperative catecholamines facilitated safe peri-anesthesia management. To our knowledge, no previous reports have described general anesthesia for surgical intervention in pediatric patients with RTD. This highlights the clinical importance of the present case, as it provides rare insight into the peri-anesthetic challenges associated with RTD.

RTD is associated with mutations of genes in the renin-angiotensin system, including angiotensinogen, renin, ACE, and angiotensin II (ATII) receptor type I, which decreases ATII activity [2]. ATII promotes sodium reabsorption in the proximal tubules and increases aldosterone production in the adrenal cortex and vasopressin production in the posterior pituitary [5]. Aldosterone increases BP by increasing sodium and water reabsorption in the distal renal tubules [6]. Vasopressin promotes reabsorption of free water in the renal collecting tubules and has a vasoconstricting effect that contributes to the regulation of fluid volume and maintenance of BP [7]. RTD is detected by a decreased amount of amniotic fluid during the prenatal period as a result of anuria, lung hypoplasia, and hypotension, which usually results in death during the neonatal period [1]. Only 18 of the 150 reported cases of RTD survived the neonatal period [3,4]. Therefore, there is limited evidence and consensus regarding the management of general anesthesia in survivors with RTD. In retrospect, the risk of profound hemodynamic collapse during the first anesthetic event was difficult to anticipate. RTD is an extremely rare condition, and no previous reports have described general anesthesia for surgical procedures in pediatric patients with RTD. Moreover, earlier episodes of hypotension in this patient had been attributed to extreme prematurity rather than an underlying defect in vascular regulation. Our patient had anuria and hypotension at birth, and RTD was diagnosed when a genetic mutation in ACE was detected. She survived the neonatal period with pharmacological maintenance of BP. Renal transplantation was also considered, but it was ultimately avoided because adequate control of urine output was achieved with fludrocortisone therapy, which had been initiated at two years of age.

Children with RTD are at risk of dehydration because of decreased production of aldosterone and vasopressin as a result of decreased ATII activity. During general anesthesia, severe hypotension can occur due to poor urine concentrating ability, preoperative dehydration, decreased preload as a consequence of positive pressure mechanical ventilation, the vasodilatory effects of anesthetic agents, and autonomic dysregulation of vasoconstriction caused by the underlying pathophysiology of RTD. Therefore, we developed a careful anesthesia plan for the second surgery that included optimizing fluid status, careful titration of anesthetics, intraoperative use of catecholamines, and ensuring reliable vascular access. In contrast to the first anesthetic event, in which inhalational induction with high-dose sevoflurane likely caused profound vasodilation and circulatory collapse, intravenous induction was selected for the second procedure. Anesthetic agents were chosen and titrated based on the underlying pathophysiology of RTD, with particular attention to preserving sympathetic tone and minimizing vasodilatory effects. Intravenous fluids were administered before the second surgery to correct preoperative dehydration associated with RTD.

Midazolam (0.3 mg/kg), ketamine (2-3 mg/kg), and fentanyl (3 μg/kg) were carefully titrated to preserve sympathetic tone and minimize hemodynamic suppression during induction. Arterial pressure and central venous lines were placed for continuous blood pressure monitoring and vasopressor administration. There is no consensus regarding which vasopressor to use in this situation. In view of the pathophysiology, vasopressin release could be impaired. Therefore, we administered vasopressin 0.33 mU/kg/min and epinephrine 0.03-0.05 μg/kg/min. Continuous administration of ATII during general anesthesia seems reasonable in children with RTD, considering their decreased ATII activity. However, we could not administer ATII to our patient because continuous ATII infusion is not available in Japan. Further studies are needed regarding the effect of administration of ATII in children with RTD during the peri-anesthesia period.

Children with RTD can become severely dehydrated during general anesthesia, leading to life-threatening hypotension. Therefore, clinicians must carefully evaluate and optimize the patient’s fluid volume throughout the peri-anesthesia period. However, there is no gold standard test for dehydration. Preoperative vitals can help to assess dehydration, but children with RTD who survive the neonatal period may have normal or abnormally low BP [3]. Thus, preoperative BP may not be a reliable indicator of dehydration in these children. As a result, careful physical examination and evaluation of laboratory tests, including vital signs, skin turgor, capillary refill time, urine output, serum BUN, urine specific gravity, and the ratio of the diameter of the inferior vena cava/aorta, are essential [8]. In our case, BP was 109/51 mmHg at the time of the first attempt to perform general anesthesia. However, HR and BUN were elevated at 132 bpm and 31.7 mg/dL, respectively. After that first attempt, the patient was started preoperatively on desmopressin 360 µg/day to optimize her fluid status. At the time of the second anesthesia, she had an HR of 76 bpm and a BUN of 11.5 mg/dL preoperatively, both of which were in the normal range. Although preoperative urine output was not recorded before either anesthetic event, it may have served as an important indicator for predicting dehydration and should ideally have been assessed. In this context, urine specific gravity can serve as a practical indicator of impaired urinary concentrating ability and hydration status in patients with nephrogenic diabetes insipidus associated with RTD. In our patient, urine specific gravity was below the detection limit before the first anesthetic event, whereas it was measurable during the second surgery, suggesting improved fluid balance and supporting its usefulness in evaluating peri-anesthetic hemodynamic stability. Preoperative control of vascular tone by administering steroids, vasopressin, and sufficient intravascular volume via intravenous infusion of fluids might have helped to prevent hypotension in the peri-anesthesia period. Intraoperative dehydration is of concern in RTD because of decreased water reabsorption in the kidney, which results in high urine output. Dehydration can increase plasma renin activity. In our case, preoperative plasma renin activity and aldosterone levels were normal at the time of the second surgery. However, there are no clear cutoff plasma renin and aldosterone values that identify dehydration.

## Conclusions

We encountered a pediatric patient with RTD who developed life-threatening hypotension immediately after anesthetic induction. Successful peri-anesthesia management during the second surgery was achieved through careful preoperative fluid optimization, cautious titration of anesthetic agents, and continuous vasopressor support. Although RTD is exceedingly rare, this case highlights the need for meticulous planning, close hemodynamic monitoring, and proactive use of vasopressors to prevent severe circulatory collapse in children with RTD.
